# *Wolbachia* Bacteria Reside in Host Golgi-Related Vesicles Whose Position Is Regulated by Polarity Proteins

**DOI:** 10.1371/journal.pone.0022703

**Published:** 2011-07-28

**Authors:** Kyung-Ok Cho, Go-Woon Kim, Ok-Kyung Lee

**Affiliations:** Department of Biological Sciences, Korea Advanced Institute of Science and Technology, Yuseong-gu, Daejeon, Korea; Institut de Pharmacologie et de Biologie Structurale, France

## Abstract

*Wolbachia pipientis* are intracellular symbiotic bacteria extremely common in various organisms including *Drosophila melanogaster*, and are known for their ability to induce changes in host reproduction. These bacteria are present in astral microtubule-associated vesicular structures in host cytoplasm, but little is known about the identity of these vesicles. We report here that *Wolbachia* are restricted only to a group of Golgi-related vesicles concentrated near the site of membrane biogenesis and minus-ends of microtubules. The *Wolbachia* vesicles were significantly mislocalized in mutant embryos defective in cell/planar polarity genes suggesting that cell/tissue polarity genes are required for apical localization of these Golgi-related vesicles. Furthermore, two of the polarity proteins, Van Gogh/Strabismus and Scribble, appeared to be present in these Golgi-related vesicles. Thus, establishment of polarity may be closely linked to the precise insertion of Golgi vesicles into the new membrane addition site.

## Introduction

*Wolbachia pipientis* are maternally inherited symbiotic bacteria that are widespread among most insects including laboratory stocks of *Drosophila melanogaster*, as well as filarial nematodes and crustaceans [1]–[4]. *Wolbachia* belong to the Richettsial family responsible for the deadly human diseases such as typhus, Rocky Mountain spotted fever, and Q fever, but themselves are not involved in any known human diseases [5]. *Wolbachia* bacteria are best known for their ability to induce reproductive alterations in hosts such as male killing, feminization, parthenogenesis, and cytoplasmic incompatibility, all of which result in increased number of infected female offspring and thereby helping vertical transfer of *Wolbachia* [6]. These reproductive alterations may promote speciation in extreme cases. Because of these intriguing properties, *Wolbachia* have been extensively studied for entomology, agriculture and evolution.

Despite *Wolbachia*'s unique role in host reproduction and physiology, their underlying cellular mechanisms are yet to be addressed. Studies with electron microscopy have revealed that *Wolbachia* bacteria are strictly present in vesicular structures in the cytoplasm of host cells [7], [8]. These *Wolbachia* vesicles are attached to astral microtubules near centrosomes by short electron-dense bridges, and their centrosomal localization is dependent on microtubules but not actin [7]. *Wolbachia* bacteria are enclosed within three layers of membranes: the outer layer is host origin and two inner layers are bacterial cell wall and bacterial plasma membrane [9]. Since parasitic bacteria and enveloped mammalian viruses often utilize a variety of subcellular organelles such as endoplasmic reticulum and Golgi apparatus during their life cycles [10]–[12], *Wolbachia* may also be present in a host organelle that can aid the replication and propagation of *Wolbachia*. Identification of this host organelle is critical for understanding the *Wolbachia*'s ability in changing host physiology.

We report here that *Wolbachia* reside in a group of Golgi-related vesicles. These Golgi-related vesicles distinctly localized near the site of membrane biogenesis in the embryo cortex, and appeared to contain two polarity proteins, Van Gogh/Strabismus (Vang hereafter) and Scribble (Scrib) as well as cis-Golgi GM130 protein. Furthermore, *Wolbachia* vesicles were mislocalized in mutant embryos defective in cell/planar polarity genes such as *discs-large (dlg)*, *Van Gogh (Vang)/strabismus (stbm)*, *frizzled (fz)* and *dishevelled (dsh)*. These observations raise an interesting possibility that *Wolbachia* may mark the unique group of Golgi vesicles linked to membrane biogenesis. The additional finding that localization of *Wolbachia* vesicles is regulated by genes involved in cell/tissue polarity also provided a surprising new potential activity for these polarity genes in Golgi localization.

## Results

It has been known that majority of fly laboratory strains is infected by *Wolbachia.* We have previously reported that numerous polyclonal antisera generated against fusion proteins expressed in *E. coil* exhibit cross-reactivity toward *Wolbachia* proteins in immunocytochemisitry, because of impurity in the antisera that have reactivity to *E. coli* proteins and also to the related *Wolbachia* proteins [13]. *Wolbachia* appear as vesicular structures with these antisera, and these false vesicular patterns can be avoided by using *Wolbachia*-free laboratory strains [13].

During the course of this previous study, we discovered a link between *Wolbachia* and Golgi-related vesicles, which is a focus of this report. To detect *Wolbachia*, we used three antisera, anti-Vang antisera [14], anti-Stardust (Sdt) antisera [15] and anti-Dlg antisera [16]. These antisera along with DNA markers recognized *Wolbachia* with higher specificity than anti-Hsp60 (cloneLK2, Sigma) or the *Wolbachia-*specific antibody [17] (data not shown).

### *Wolbachia* bacteria are present in Golgi-related vesicles

**Figure 1:**
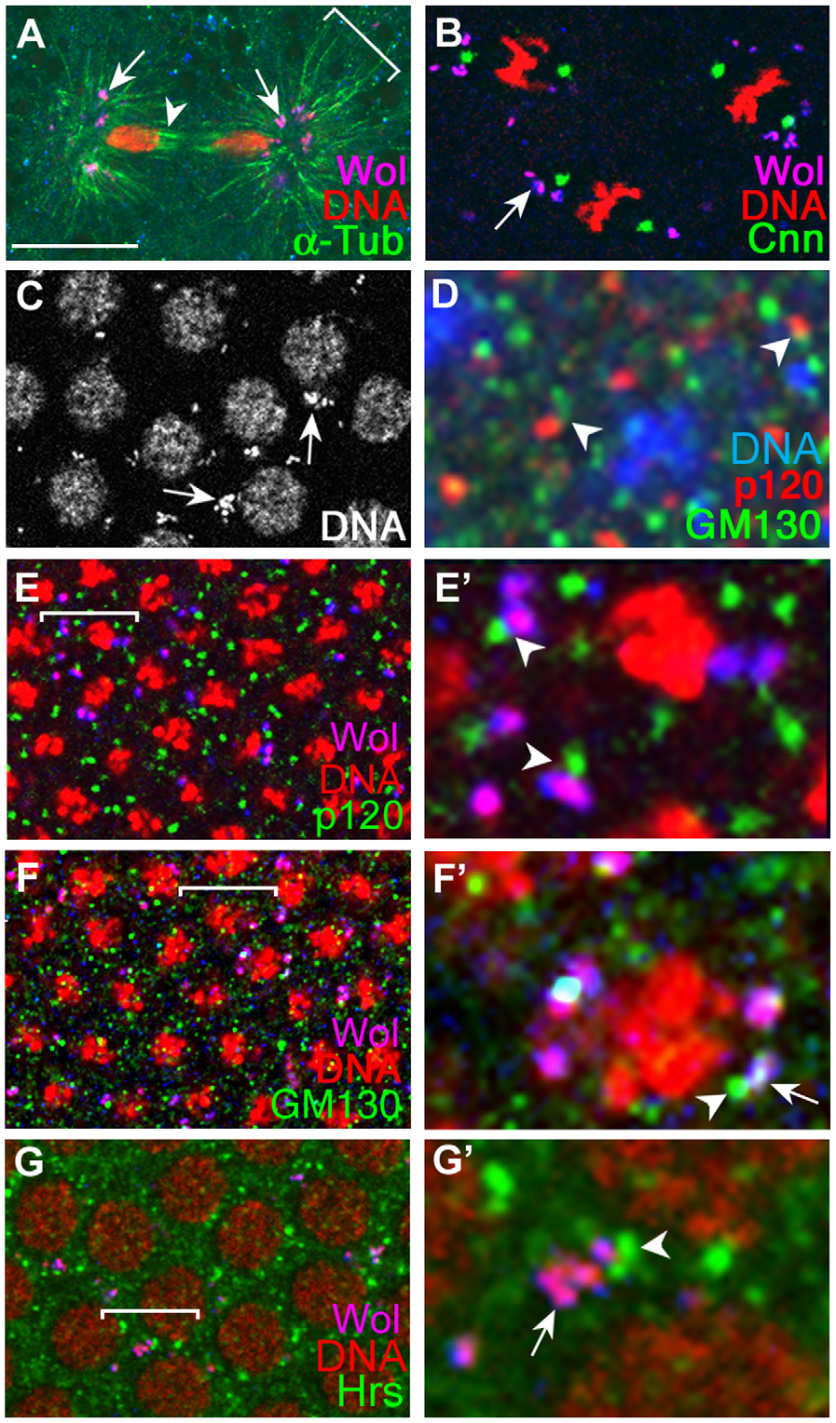
*Wolbachia* bacteria are present in Golgi-related vesicles. *Wolbachia*-infected *CS* embryos were used to generate all images except (D). (D) was obtained with *Wolbachia*-free *CS* embryos. *Wolbachia* in all images except (A) were visualized with anti-Vang antisera, while those in (A) were visualized with anti-Sdt antisera. *Wolbachia*, recognized by antisera (blue) and DNA marker (red), appear as pink. In these images, structures appeared as blue do not contain DNA and should be considered to have endogenous Sdt (A) or Vang (B-G except D). DNAs in A, B, C and G were visualized with propidium iodide, and DNAs in D, E, and F were visualized with Toplo-3. (A) In a preblastoderm stage embryo, *Wolbachia* vesicles (arrows) are attached near the minus ends of the astral microtubules (bracket) but not the polar microtubules (arrowhead). (B) A blastoderm stage embryo shows *Wolbachia* localization near centrosome (arrow). (C) *Wolbachia* vesicles are perinulclear during interphase (arrows). (D) GM130 and p120 are present in separate vesicles in *Wolbachia-*free *CS* embryos during mid-cellularization. They sometimes are present in the two adjacent vesicles (arrowheads). (E,F) In *Wolbachia*-infected CS embryos during mid-cellularization, p120-containing vesicles are physically separated from *Wolbachia* vesicles, but are in proximity (arrowheads) (E). *Wolbachia* vesicles either are juxtaposed to GM130-containing vesicles (arrowheads) or contain GM130 proteins (arrow) (F). (G) In *Wolbachia*-infected *CS* embryos, some *Wolbachia* vesicles are in proximity with Hrs vesicles (arrow), but did not contain Hrs (arrowhead). Portions marked with brackets in E, F, and G are magnified in E', F' and G'. Scale bar: A,C,E,F,G, 10 µm; B, 4.4 µm; D,E',F',G', 2.5 µm.We then examined the pattern of GM130 and p120 in *Wolbachia*-infected *CS* embryos. The *Wolbachia* vesicles rarely contained p120 protein (<1%; 2/228), but 46% of *Wolbachia* vesicles located close to the p120 vesicles (106/228) (Figure 1E and 1E'). In contrast, 17% of *Wolbachia* vesicles contained GM130 protein (40/238), and 76% were juxtaposed to GM130-containing vesicles (180/238) (Figure 1F and 1F'). These data suggest that *Wolbachia* bacteria reside in a type of Golgi vesicles that are closely related to cis-Golgi. We also found that *Wolbachia* were not present in endosomes, using an antibody against Hepatocyte growth factor-regulated tyrosine kinase substrate (Hrs) that is present in endosomes [25] (Figure 1G). In conjunction with the previous report that *Wolbachia* are absent in mitochondria [26], we concluded that *Wolbachia* are present in a group of cis-Golgi related vesicles.

*Wolbachia* bacteria are present in membrane-bound vesicular structures that are attached to astral microtubules near centrosomes ([7]: Figures 1A and 1B) and are mostly perinuclear during interphase (Figure 1C). Since such localization patterns are reminiscent of mammalian Golgi apparatus, we reasoned that *Wolbachia* bacteria may be present in host Golgi vesicles. To test this possibility, we utilized two Golgi markers, GM130 and p120. GM130 is a tightly associated peripheral cis-Golgi protein that is involved in Golgi ribbon formation as well as mitotic Golgi fragmentation in mammalian cells [18]–[21]. p120 is proposed as a fly homolog of rat MG-160, a sialoglycoprotein of the medial Golgi cisternae [22]–[24]. It has been shown that GM130 and p120 are present in the two juxtaposed, but clearly distinct vesicles in fly imaginal discs during the third-instar larval stage (See Figure 1G in [24]), suggesting that the cis- and the medial-Golgi are physically distinguishable in flies. We observed in fly embryos that GM130 and p120 were sometimes present in the juxtaposed vesicles but rarely in the same vesicle, indicating that the cis- and medial Golgi units are spatially separated from each other in both embryos and larvae (Figure 1D).

### *Wolbachia* vesicles are concentrated near the site of membrane biogenesis

**Figure 2:**
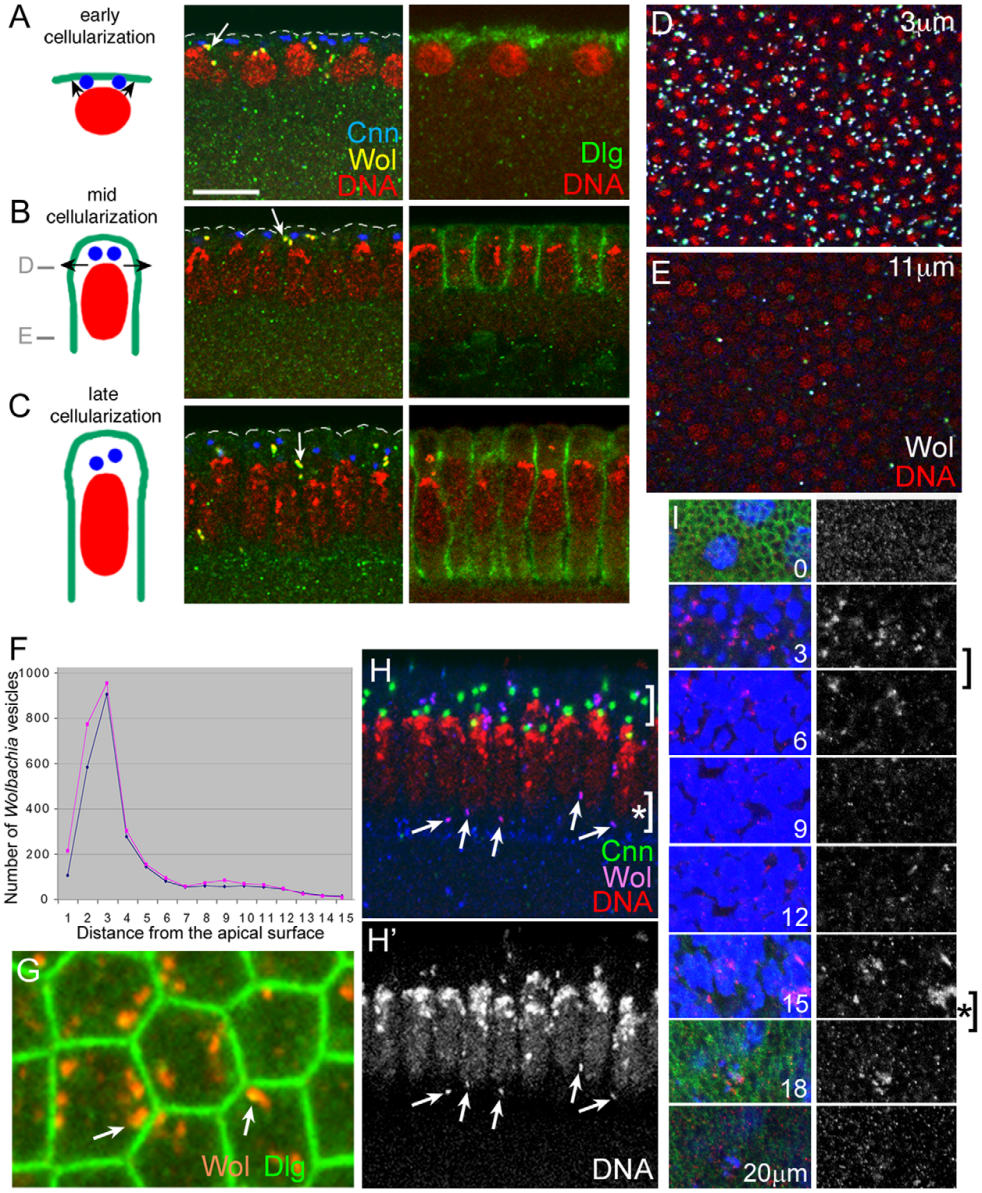
*Wolbachia* vesicles are enriched near new membrane addition sites. For detecting *Wolbachia*, anti-Dlg antisera was used for A, B, and C, anti-Vang antisera were used for H and I, while both anti-Vang and anti-Dlg antisera were used for G, and both anti-Vang and anti-Sdt antisera were used for D and E. DNA was visualized with propidium iodide in all images except G and I. (A,B,C) **Left**, At the onset of cellularization, new membrane addition occurs at the apical region (arrows in A). At mid-cellularization stage, major membrane addition site is sub-apical region (arrows in B). At the end of cellularization, elongated nuclei are separated by newly formed cell boundary (C). The blue dots represent centromes, the red ovals are nuclei, and the green lines are membranes. This diagram is based on information from Lecuit and Wieschaus [27]. **Middle**, *Wolbachia* vesicles are indicated with arrows. Centrosomes visualized with Centrosomin antibody (blue) are present at the apical region. **Right**, Dlg (green) is present at the membrane. Nuclei are initially round and become elongated as the cellularization proceeds (red). (D–F) Among the 15 tangential sections with 1 µm interval, the *Wolbachia* vesicles are most enriched at 3 µm from the apical surface (D), and are almost absent at the basal level at mid-cellularization stage (E). The planes of confocal sections, D and E, are indicated in B with grey bars. Number of *Wolbachia* vesicles in these 15 tangential sections was counted with NIH Image J program (F). The number of *Wolbachia* vesicles was separately counted from the data obtained with two antisera that can recognize *Wolbachia* [13]. (G) *Wolbachia* vesicles are located close to the plasma membrane (arrows). (H) A cross-section of embryo cortex at the end of cellularization. *Wolbachia* vesicles are localized not only in the sub-apical region (bracket) but also in the basal region (arrows, bracket with asterisk). (I) *Wolbachia* vesicles are visualized by serial tangential sections of epithelial cells in a wing disc. Density of *Wolbachia* vesicles is highest around 2–6 µm from the apical surface (bracket), and a minor fraction of *Wolbachia* is present near the basal position (bracket with an asterisk). Dlg was visualized as a membrane marker (green). *Wolbachia*, stained with both DAPI (blue) and anti-Vang antibody (red), appear as pink. Scale bar: A–C, 10 µm; D,E,H,I, 14 µm; G, 3.7 µm.

Previous studies have shown that *Wolbachia* vesicles are concentrated in the cortical layer and also scattered in the entire cytoplasm of newly laid embryos. As the embryo further develops to syncytial blastoderm stage, most *Wolbachia* vesicles become localized to the cortex along with nuclei and centrosomes that have migrated from the embryo interior to the cortex [7], [8]. During the subsequent cellularization stage, we found that *Wolbachia* vesicles became more narrowly concentrated in the sub-apical region of the cortex (Figures 2A, 2B and 2C): approximately 80% of *Wolbachia* vesicles (2406/2960) were concentrated in the 5 µm span of apical region. The highest percentage (∼32%) of *Wolbachia* vesicles (955/2960) was at ∼3 µm from the apical surface of the cellularizing embryo (Figures 2D, 2E and 2F). This region with highest percentage of *Wolbachia* vesicles precisely coincide with the new membrane addition site that is located in between the apical and the basolateral regions, as identified by Lecuit and Wieschaus [27]. They showed that membrane addition occurs only at the sub-apical region of plasma membrane during mid-cellularization. Further, these *Wolbachia* vesicles were concentrated near the newly forming cell boundary (Figure 2G). These data raise a possibility that *Wolbachia* selectively reside in a special group of Golgi-related vesicles that is involved in membrane biogenesis of newly forming epithelial cells.

At the end of cellularization stage, a minor fraction of *Wolbachia* vesicles was found near the region between the membrane front and the growing lateral membrane (arrows in Figure 2H). This region corresponds to the basal adherens junction, whose integrity is essential for the growth of the plasma membrane [28], [29]. Because *Wolbachia* vesicles were not found near the basal junction during the mid-cellularization when the extensive membrane biogenesis occurs (Figure 2F), localization of *Wolbachia* vesicles near the basal adherens junction may be a unique feature of established epithelial cells. We therefore examined whether the epithelial cells in wing imaginal discs, another example of established epithelial cells, also have *Wolbachia* vesicles near basal adherens junction. As shown in Figure 2I, majority of *Wolbachia* vesicles was enriched sub-apically at the region ∼2–6 µm from the apical surface of wing epithelial cells, but a minor fraction of them was also found near the basal adherens junction. These data show the similarity between the embryo and larval epithelial cells in terms of *Wolbachia* localization, and suggest that similar Golgi-related vesicles are present near the membrane addition site and the basal adherens junction. Further studies are required to reveal the role of these vesicles in the two different membrane sites.

### *Wolbachia*-containing vesicles are mislocalized in several polarity mutant embryos

**Figure 3:**
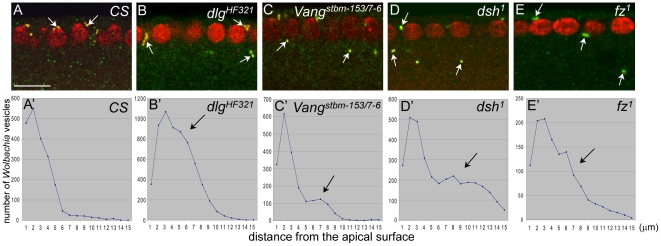
Apical positioning of *Wolbachia* vesicles is disrupted in *dlg, Vang, dsh* and *fz* mutant embryos during early to mid cellularization. *Wolbachia* were visualized with anti-Vang antisera and propidium iodide. (A–E) *Wolbachia* vesicles are marked with arrows. Note that some *Wolbachia* vesicles in the mutant embryos are present in the basal location (B–E) compared to *CS* embryo (A). (A'–E') The numbers of *Wolbachia* vesicles counted with Image J program were plotted. Arrows indicate the region where abnormally high level of *Wolbachia* along the apical-basal position.

If *Wolbachia* are indeed present in a special group of Golgi vesicles participating in membrane growth, *Wolbachia* could be used as a marker for these Golgi vesicles. We have previously reported that Dlg and its partner Vang are involved in new membrane growth, in addition to their well-studied functions in apical-basal cell polarity and planar cell polarity (PCP) [14], [30]–[34]. Thus, we reasoned that Golgi vesicles involved in membrane growth might be mislocalized in *dlg* and *Vang* mutant embryos. To test this, we examined the localization pattern of *Wolbachia* in these mutant embryos. The temperature sensitive *dlg^HF321^* embryos obtained from the homozygous *Wolbachia*-infected *dlg^HF321^* parents were cultured at the restrictive temperature (25°C) in order to obtain partial loss of function *dlg* phenotype. *Vang^stbm-153^* and *Vang^stbm-7-6^* embryos were obtained from the crosses between homozygous mutant adults. Unlike other *Vang* null mutant such as *Vang^stbm-6^*, both *Vang^stbm-153^* and *Vang^stbm-7-6^* are hypomorphs that can produce homozygous embryos with varying degrees of hatching rate: at 25°C, 82% of *Vang^stbm-153^* embryos and 20% of *Vang^stbm-7-6^* can hatch when 99% of wild type embryos can hatch [31]. *Vang^stbm-153^* allele contains a frameshift mutation that can generate a truncated protein of 205 amino acid residues [31]. The molecular lesion of *Vang^stbm-7-6^* has not been identified (Flybase). Interestingly, *Vang^stbm-153^* flies can be maintained as a healthy homozygous stock, suggesting that they may contain a partially functional Vang protein. Cross-sections of these *Vang* embryos revealed that *Wolbachia* vesicles were frequently located below the cortex in these *dlg^HF321^*, *Vang^stbm-153^* and *Vang^stbm-7-6^* embryos, unlike the *Wolbachia* vesicles in wild type embryos that were strictly present in the sub-apical region of the cortex (Figures 3A–3C).

The *Vang* gene is shown to genetically interact with other PCP genes such as *dsh* and *fz*, and Vang protein also physically interacts with Dsh and Fz [32], [35], [36]. Potential involvement of Vang in Golgi vesicle localization raised a possibility to us that other PCP proteins may also play a role in apical localization of Golgi vesicles in cellularizing embryos. To test this notion, *dsh^1^* and *fz^1^* embryos infected with *Wolbachia* were examined. As shown in Figures 3D and 3E, a significant fraction of *Wolbachia* vesicles in *dsh^1^* and *fz^1^* embryos was also found in embryo interior during cellularization, which was further confirmed by quantitative analysis (Figures 3A'–3E'). Mutations in non-PCP genes such as *syntaxin* did not result in mislocalization of *Wolbachia* vesicles (Figure S1). It appeared that the PCP proteins may play previously unidentified roles in localization of Golgi vesicles important for membrane biogenesis.

### Scribble and Vang are enriched in Golgi vesicles

**Figure 4:**
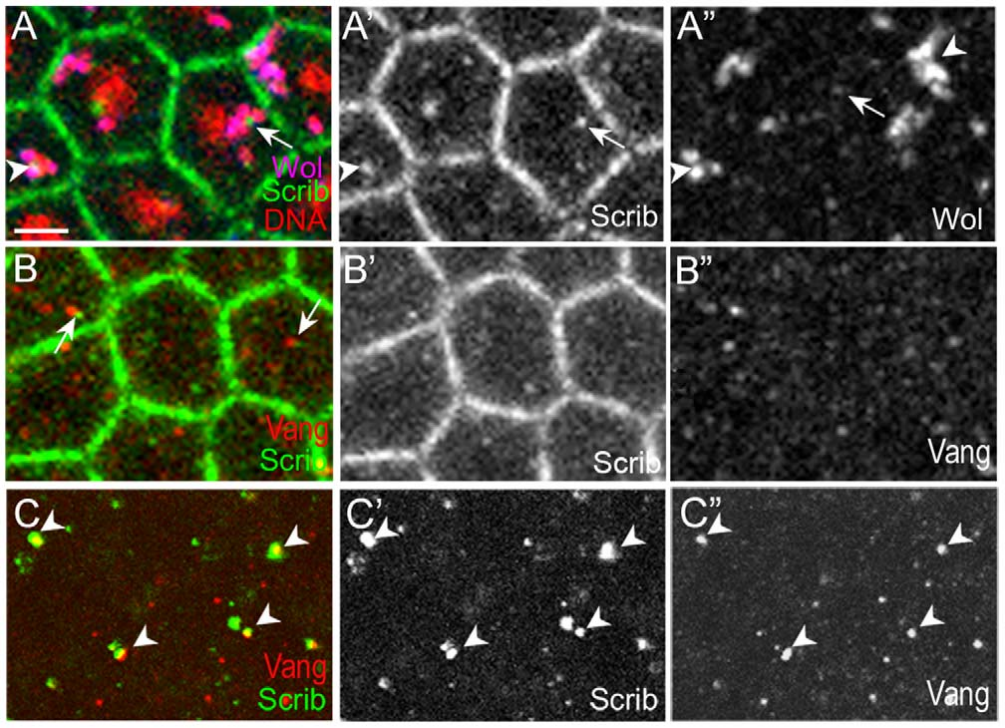
Scrib is present in vesicles that are juxtaposed to *Wolbachia* vesicles. (A) In the cortex of cellularizing *Wolbachia*-infected embryos, Scrib is present in both cell boundary and cytoplasmic vesicles. Scrib vesicles either contain *Wolbachia* (arrowhead) or are surrounded by *Wolbachia* vesicles (arrow). *Wolbachia* were identified with anti-Vang antisera (blue) and propidium iodide (red). Therefore, *Wolbachia* appear as pink, while endogenous Vang appears as blue in (A). In A”, the strong staining (arrowheads) indicates *Wolbachia*, but the weak staining indicates endogenous Vang (arrow). (B) In the cortex of cellularizing *Wolbachia*-free embryos, Vang and Scrib are present in vesicles that are sometimes juxtaposed (arrows). (C) In the embryo interior of cellularing *Wolbachia*-free embryos, large-sized Vang-containing vesicles all contain Scrib (arrowheads). Scale bar: 3.3 µm.

Control of *Wolbachia* vesicle localization by PCP proteins suggests that some of these PCP proteins might actually function in the Golgi vesicles. We previously reported that Vang and GM130 frequently colocalize to the same vesicles in both fly embryos and human TE85 cells [14]. We extended this study to Fz and Dsh proteins, but these proteins were either expressed at a very low level or devoid of any distinct patterns, thereby making it difficult to draw any conclusion. We then turned our attention to Scrib, because Vang and Scrib not only genetically interact but also show direct physical interaction [37]–[39]. Fly Scrib is essential for establishment of apico-basal polarity and cooperates with Vang in PCP establishment [39], [40]. Scrib is involved in many cellular functions related to PCP genes such as hair cell orientation and convergent extention in mammals [38], [40], [41]. Interestingly, *Wolbachia* vesicles in embryonic neuroblast cells are shown to concentrate near the apical membrane where Scrib is enriched [42]. We found that, in addition to the apical membrane of the cellularizing embryo, Scrib was present in cytoplasmic vesicles that either contained *Wolbachia* or tightly surrounded by *Wolbachia* vesicles (Figures 4A–A″).

Presence of Scrib in vesicular structures prompted us to examine whether Scrib and Vang colocalize in the same vesicles in the sub-apical region of *Wolbachia*-free embryos. Any structure recognized by anti-Vang antibody should be considered to contain endogenous Vang protein in *Wolbachia-*free embryos, based on the specificity of anti-Vang antibody (Figure S2) [14]. As shown in Figure 4B, Scrib and Vang were sometimes present in the juxtaposed vesicles in the sub-apical region during mid-cellularization. More significant colocalization was observed in the vesicles in the embryo interior: all large-sized Vang-containing vesicles also contained Scrib (Figure 4C).

**Figure 5:**
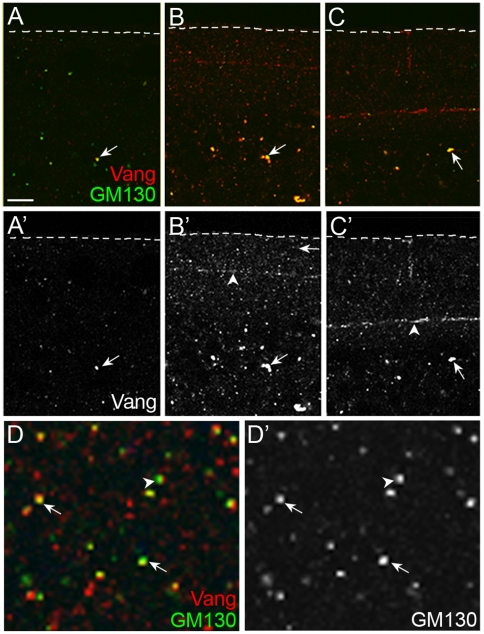
Vang and GM130 are present in same vesicles or juxtaposed vesicles. (A, B, C) Vesicles containing Vang and GM130 in immediately after egglaying (A), during mid-cellularization (B), and late cellularization (C) are indicated with arrows. Arrowheads in B' and C' indicate the furrow front where Vang is enriched. (D) A tangential section was obtained around 3.5 µm from the apical surface of the embryo in (B) (white line). Arrows indicate the vesicles containing both Vang and GM130, and the arrowhead indicates two juxtaposed vesicles. Scale bar: A–C, 10 µm; D, 2.5 µm.

We then examined the relationship between the Vang-containing vesicles and GM130 in more detail. In newly laid embryos, Vang-containing vesicles were small and rarely contained GM130 (Figure 5A). In contrast, the number and the size of Vang-containing vesicles in the embryo interior noticeably increased during the following mid-cellularization stage when the extensive membrane growth occurs. Furthermore, almost all medium to large-sized Vang-containing vesicles in the embryo interior also contained GM130 (Figure 5B). Another finding was that the number of these large Vang vesicles decreased significantly at the end of cellularization when there is no further membrane growth (Figure 5C). This suggests that these Vang-containing vesicles may be an intermediate form of Golgi vesicles that is prerequisite for the final Golgi vesicles involved in membrane biogenesis during cellularization.

**Figure 6:**
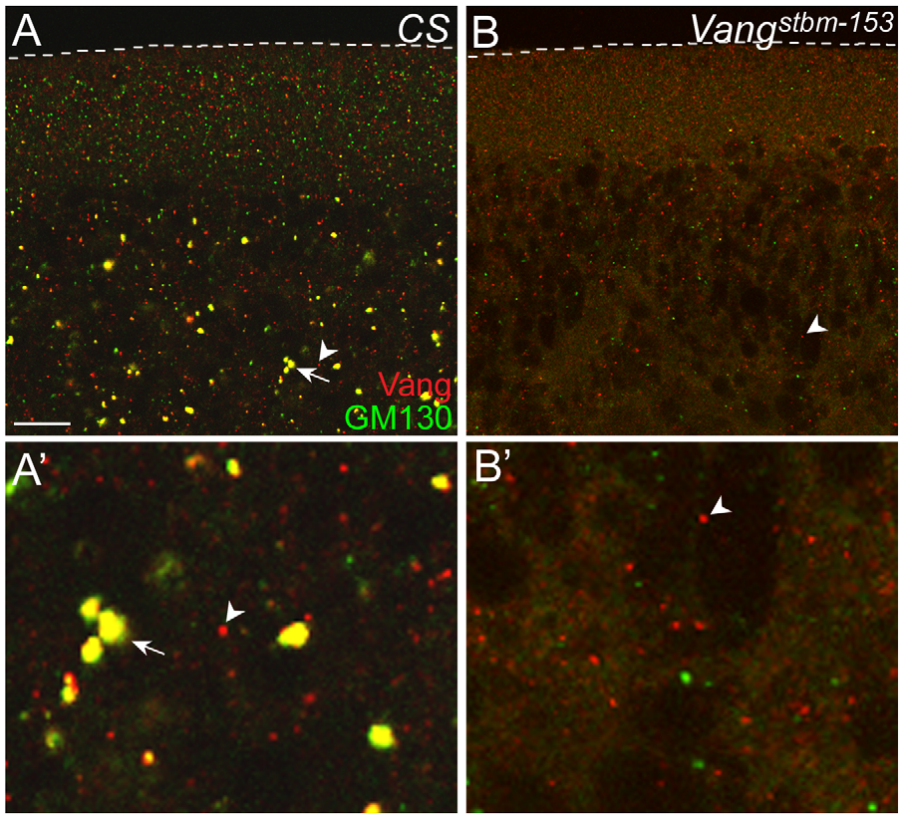
The vesicles that contain both Vang and GM130 are absent in *Vang* mutant embryos. While *Wolbachia*-free *CS* embryo during mid-cellularization contains numerous vesicles that contain both Vang and GM130 (arrow in A), *Wolbachia*-free *stbm^153^* embryo during mid-cellularization does not have such vesicles (B). Arrowheads indicate the vesicles containing only Vang. The regions with arrowheads in A and B are magnified in A' and B', respectively. Scale bar: A,B, 10 µm.

We have shown that *Wolbachia* are either present in the GM130-containing vesicles or in the vesicles juxtaposed to GM130-containing vesicles in the sub-apical region of embryos during mid-cellularization (Figures 1F and 1F'). Since anti-Vang antisera have cross-reactivity to *Wolbachia,* it is not possible to check whether Vang is also present in *Wolbachia* vesicles. If Vang is indeed present in Golgi vesicles that are harbored by *Wolbachia*, we reasoned that Vang should be present in the same vesicles with GM130 at the sub-apical region in the cellularizing embryo. As shown in Figures 5D and 5D', we found that all large GM130-containing vesicles either contained Vang or juxtaposed to Vang-containing vesicles in the *Wolbachia-*free embryo. This strongly suggests that the Golgi vesicles containing both GM130 and Vang may harbor *Wolbachia* or may be present in the vesicles juxtaposed *Wolbachia* vesicles. Thus, there is a possibility that Vang, Scrib and GM130 are present in the same Golgi vesicles, although further study is necessary to provide direct evidence. We then tested whether these Vang and GM130-containing vesicles are affected in mutant embryos that are defective in membrane biogenesis. Unlike in wild type embryos, such medium to large sized vesicles containing Vang were not detected in the embryo interior of *Vang^stbm-153^*, *Vang^stbm-153^/Vang^stbm-7-6^,* and *dlg^HF321^* embryos except only in small vesicles (Figures 6 and Figure S3). This suggests that both Vang and Dlg may be involved in generation or maturation of these large Golgi vesicles.

## Discussion

*Drosophila* Golgi system is similar to mammalian Golgi system in its structure and function, and clearly displays several cisternae per stack that are polarized with cis and trans faces [43]. Although these fly Golgi vesicles are functionally diverse and can be distinguished by differences in glycosylation, they are detected as scattered dotty structures by most fly Golgi markers with confocal microscopy because their cisternae are not interconnected, and their size is less than half than that of mammalian counterpart [24], [43]. Here we report that *Wolbachia* bacteria specifically reside in a special group of Golgi-related vesicles that may be functionally linked to membrane biogenesis. This makes *Wolbachia* an attractable marker for detecting functional fly Golgi vesicles. *Wolbachia* being in Golgi is also consistent with the previous report that *Wolbachia* are present in cytoplasmic vacuoles that are associated with astral microtubules and whose outmost membrane is host origin ([7], Figure 1A).

We found a special relationship between *Wolbachia* and astral microtubules: all *Wolbachia* vesicles localized near the minus-ends of microtubules but not the plus-ends of microtubules (Figure 1A). As the Golgi apparatus of mammalian cells is also shown to localize to the minus ends of microtubules, association between Golgi and the minus ends of microtubules may be a universal phenomenon [44], [45]. Same conclusion could be drawn from analysis of literatures on the localization patterns of *Wolbachia* and microtubules in developing fly oocytes. During mid-oogenesis when microtubules play an essential role for axis formation, microtubule density decreases at the posterior region but increases at the anterior region of the oocyte [46]. At this time, minus-ends of microtubules are precisely concentrated at the anterior pole of oocyte, where *Wolbachia* vesicles are concentrated [26]. After this stage, both *Wolbachia* and minus-ends of microtubules become dispersed throughout entire oocyte [26]. These indicate that *Wolbachia* and minus-ends of microtubules colocalize during this critical stage, and polarization of Golgi vesicles may be important for delivering the axis determinants from the nurse cells to the right regions in the developing oocyte. Another link between *Wolbachia* and the minus-ends of microtubules is the mislocalization of *Wolbachia* in *dynein* mutants; anterior enrichment of *Wolbachia* in developing oocytes is disrupted when the *dynein* gene coding for a microtubule minus-end directed motor is mutated. In contrast, mutations in the *kinesin* gene that codes for a microtubule plus-end directed motor, do not cause any changes in *Wolbachia* localization in *Drosophila* oocyte [26], [47], [48].

An important clue for identifying the function of these Golgi vesicles came from the comparison of *Wolbachia* localization in wild type and various polarity mutant embryos during cellularization. *Wolbachia* vesicles were greatly enriched near the new membrane addition site in wild-type embryos, implying their potential involvement in membrane biogenesis (Figure 2F). Fusion of Golgi vesicles onto the pre-determined membrane addition site leads to the addition of both membrane lipids and associated proteins to the right domain of the plasma membrane, which ensures not only the membrane growth but also the establishment of cell and tissue polarity. Mislocalization of *Wolbachia* vesicles in embryos defective in polarity genes such as *dlg*, *Vang*, *fz* or *dsh*, thus indicates that these polarity genes may somehow be involved in localizing these Golgi vesicles (Figure 3).

Involvement of polarity proteins in localizing Golgi vesicles has been recently reported. Dsh is shown to control association of membrane-bound vesicles and Sec8, a vesicle-trafficking protein, in order for apical docking of basal bodies in ciliated epithelial cells [49]. Vangl2, a mammalian homolog of Vang, is also selectively sorted into COPII vesicles by Sec24b, and Vangl2 looptail point mutant proteins fail to sort into COPII vesicles and are trapped in the ER [50], [51]. Sec24b is a cargo-sorting member of the core complex that is important for formation of ER-to-Golgi transport vesicle COPII, and also genetically interacts with Scrib [51]. Furthermore, the knock-out mice mutated in *Vangl2*, *scrib*, or *sec24b* gene all show almost identical neural tube defects in addition to polarity defects [50]-[53]. Therefore, similar to Dsh, Vangl2 and Scrib may play a direct role in formation or localization of COPII vesicles instead of being just a cargo protein, and Vang and Scribble together with Sec24b may be involved in this process.

To properly localize the Golgi vesicles involved in membrane biogenesis, all the players that are involved in multiple Golgi maturation steps should sequentially act. Thus, mislocalization of *Wolbachia* vesicles may indicate that the Golgi vesicles are not fully matured, and consequently not capable of fusing to the plasma membrane. If some of the polarity proteins are the players in these Golgi maturation processes, the *Wolbachia* vesicles, indicative of the matured Golgi vesicles, would be mislocalized in mutants of the polarity genes. We found that cells in both *dlg* and *Vang* mutant embryos frequently show lack of membrane, supporting this idea [14]. Our data that the large vesicles containing both Vang and GM130 were not detected in a *Vang* mutant, also support the idea that Vang may be essential for the maturation of Golgi vesicles (Figure 6).

One of the well-studied examples of PCP is the hair polarity in the fly wing [54]. Hair formation is restricted to the distal part of each wing cell by the core PCP proteins, Fz, Dsh, Vang, and Prickle (Pk) [32], [55]–[58]. These core PCP proteins are also involved in the PCP of photoreceptor cells and embryonic denticles [31], [35], [59]–[61]. In case of the wing hairs, apical localizations of Vang and Pk in the proximal membrane and Dsh and Fz in the distal membrane in each wing cell during pupal stage are crucial for the positioning of a single distal hair. It is still largely unknown how the selective localization of these PCP proteins is achieved, but at least Fz protein appears to be delivered along the apical microtubules to the distal membrane of the hair cells [62]. The authors found that intracellular vesicles containing Fz-GFP marker preferentially move along the distally oriented apical microtubules and join the distal membrane [62]. One can imagine that the vesicles containing Vang or Pk may be preferentially delivered to the proximal membrane along the apical microtubules. Based on our data and others, we propose that these PCP proteins may play major roles in apical positioning of Golgi vesicles in either proximal or distal region of the wing cell, whose precise position is essential for cell and tissue polarity. When any one of these PCP proteins is not fully functional, proteins essential for PCP function may not be delivered to the proper location at the membrane, and consequently, both apical-basal and proximal-distal polarity would be disrupted. Taken together, these PCP proteins may not be just passively transported to the destined location at the membrane, but rather actively regulate the apical localization and the delivery of the distinct groups of Golgi vesicles.

## Materials and Methods

### Fly strains

The original *CS* strain containing *Wolbachia pipientis*, the same *CS* strain cured by tetracycline (250 µg/ml food) treatment [13], [63] as well as *Wolbachia*-infected polarity mutants were used for this study. To infect the flies with the same type of *Wolbachia*, all the flies were first treated with tetracycline for three generations to cure any resident *Wolbachia*. The male flies from treated population were then crossed with the females of the *Wolbachia*-infected balancers. The siblings that had become infected with *Wolbachia* were mated to generate a *Wolbachia*-infected line.

### Immunocytochemistry

Embryos were collected at either room temperature or 25°C, and fixed in 4% formaldehyde (methanol-free) by heptane method. We found that *Wolbachia* staining was quite strong with the anti-Vang and anti-Sdt antisera, and somewhat less with the anti-Dlg antisera [17]. Therefore, strong signals from antibody staining that also contain DNA were considered as *Wolbachia* in *Wolbachia*-infected embryos. In *Wolbachia-*free embryos, these antisera should recognize only their own endogenous proteins. For DNA staining, embryos after secondary antibody incubation were incubated with ribonuclease and then stained with propidium iodide. Alternatively, To-Pro-3 (Molecular Probes) or DAPI were used to stain nuclei.

Following antibodies were used for tissue staining: rabbit anti-Dlg [16]; mouse anti-Vang [14]; rabbit anti-Sdt [15]; rabbit anti-GM130 [64]; rabbit anti-Cnn [65]; guinea pig anti-Hrs [25]; rabbit anti-Scrib [41]; rat anti-Dsh [66]; mouse anti-Fz (1C11 monoclonal, Developmental Hybridoma Bank); mouse anti-p120 (Calbiochem); mouse anti-α-Tubulin (clone DM1A, Sigma). Fluorescent images were captured using Zeiss LSM laser-scanning confocal microscope and presented using Adobe Photoshop.

### Quantitative analysis of *Wolbachia* vesicles

*Wolbachia* vesicles were visualized with propidium iodide as well as two antisera, anti-Stardust (rabbit) and anti-Vang (mouse) antisera. The vesicular structures detected with all three markers were counted as *Wolbachia* vesicles. In order to obtain quantitative data, series of confocal sections taken along the apical-basal axis were processed with NIH Image J program. Since the density of *Wolbachia* varied from embryos to embryos, we presented data obtained from a representative embryo in Figure 3, instead of averaging the number of *Wolbachia* from different embryos along the apical-basal axis. Cross-sections of at least 20 embryos were examined for each mutation, 3 representative embryos were chosen for serial tangential sections, and one of them was presented in Figure 3.

## Supporting Information

Figure S1 ***Wolbachia*** **are not mislocalized in** ***sys^ts3^*** **embryos.** *Wolbachia* in a *sys^ts3^* embryo are apically localized (arrows). *Wolbachia* are detected with anti-Vang antisera and propidium iodide.
(TIF)

Figure S2 **Anti-Vang antisera are specific to Vang protein.** (A) Vang protein is overexpressed in the wing disc of offspring obtained from the cross between *UAS-Vang* and *patched-Gal4* parents, and was detected with anti-Vang antisera precleared with agarose-bound GST protein. (B) Same tissues were incubated with anti-Vang antisera precleared with agarose-bound GST-Vang protein. Same regions in the wing pouch were shown.
(TIF)

Figure S3 **The Vang-containing vesicles are absent in** ***Vang*** **and** ***dlg*** **mutant embryos.** All three embryos are *Wolbachia*-free, and the black and white images show the numerous medium to large sized Vang vesicles in *CS* embryos (A), only small Vang vesicles in *Vang^stbm-153^* and *Vang^stbm-153^ Vang^stbm-7-6^* embryos (B, C), and lack of Vang vesicles in *dlg^HF321^* embryos (D). Images in A and B in this figure and the ones in Figures 6A and B are generated from the same original images.
(TIF)
