# Supplementary figures and images for: Wolbachia Bacteria Reside in Host Golgi-Related Vesicles Whose Position Is Regulated by Polarity Proteins
Source: PLoS One. 2011 Jul 28;6(7):e22703. doi: 10.1371/journal.pone.0022703 (PMC3145749; doi:10.1371/journal.pone.0022703)

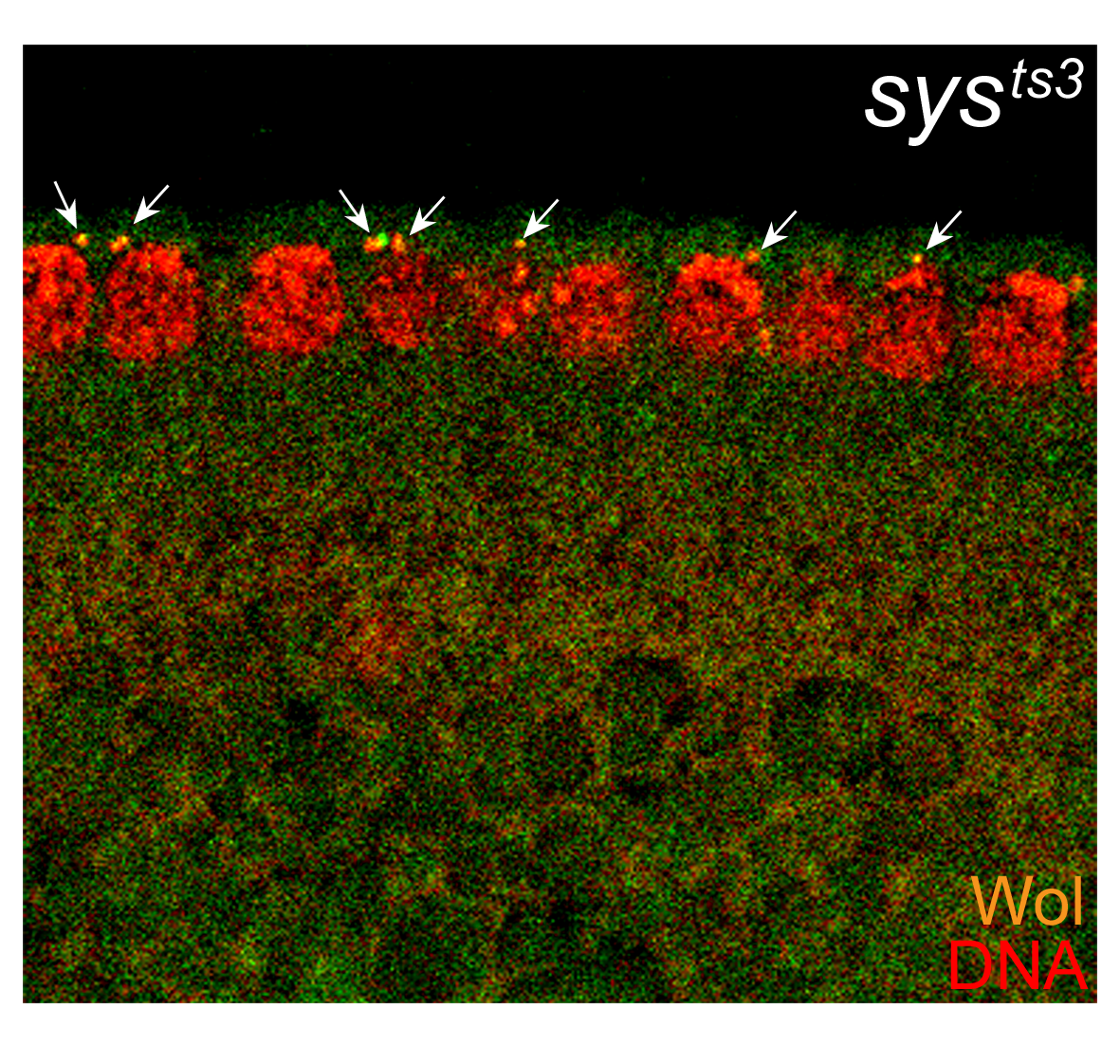

Supplement: Figure S1 — Wolbachia are not mislocalized in systs3 embryos. Wolbachia in a systs3 embryo are apically localized (arrows). Wolbachia are detected with anti-Vang antisera and propidium iodide. (TIF) [file pone.0022703.s001.tif]

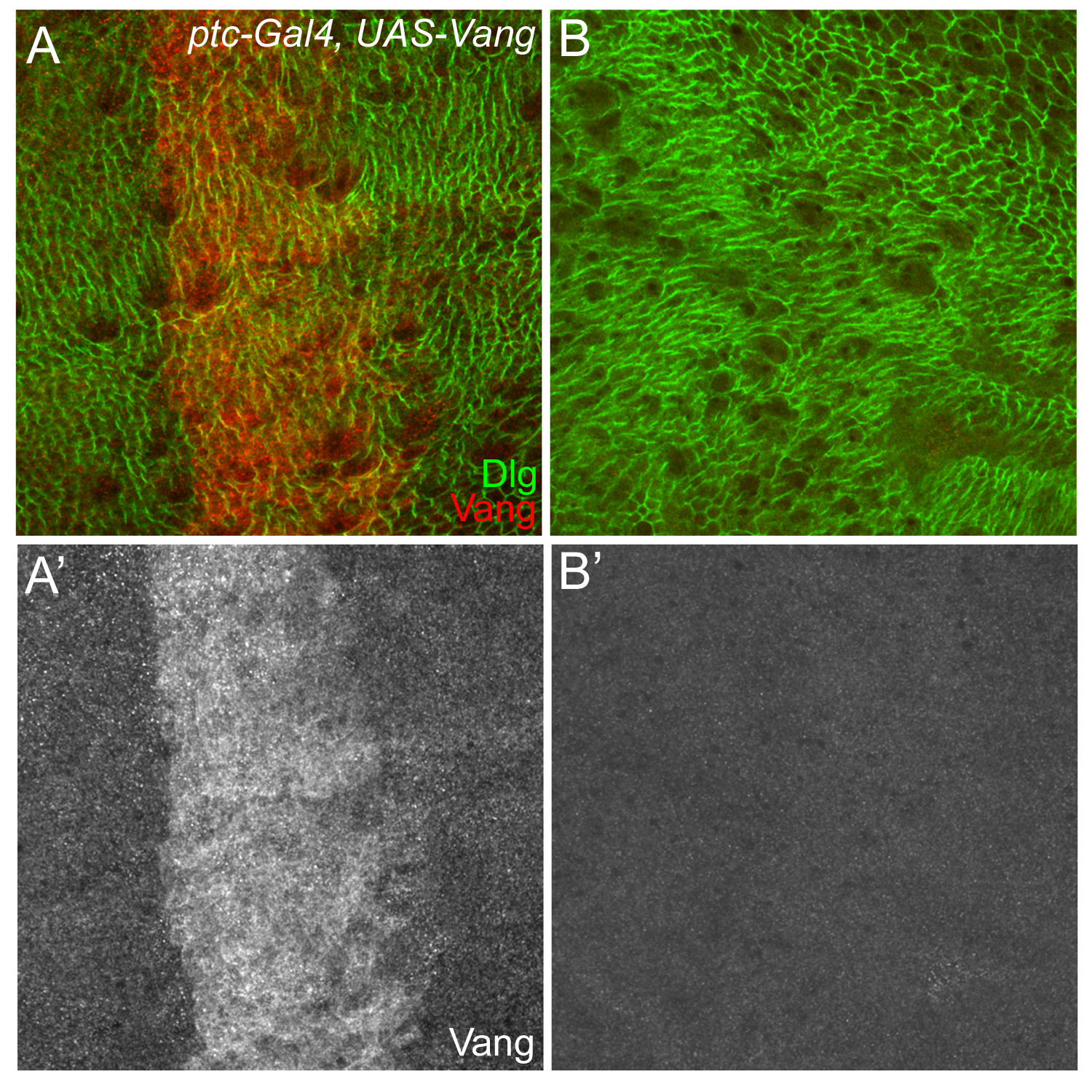

Supplement: Figure S2 — Anti-Vang antisera are specific to Vang protein. (A) Vang protein is overexpressed in the wing disc of offspring obtained from the cross between UAS-Vang and patched-Gal4 parents, and was detected with anti-Vang antisera precleared with agarose-bound GST protein. (B) Same tissues were incubated with anti-Vang antisera precleared with agarose-bound GST-Vang protein. Same regions in the wing pouch were shown. (TIF) [file pone.0022703.s002.tif]

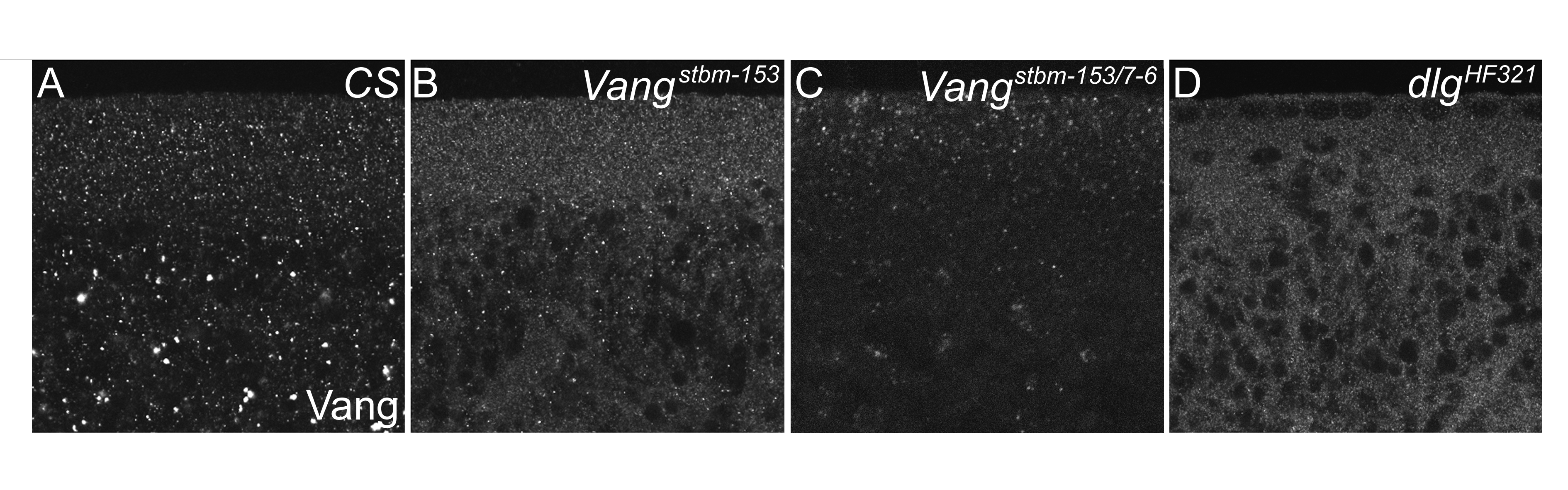

Supplement: Figure S3 — The Vang-containing vesicles are absent in Vang and dlg mutant embryos. All three embryos are Wolbachia-free, and the black and white images show the numerous medium to large sized Vang vesicles in CS embryos (A), only small Vang vesicles in Vangstbm-153 and Vangstbm-153 Vangstbm-7-6 embryos (B, C), and lack of Vang vesicles in dlgHF321 embryos (D). Images in A and B in this figure and the ones in Figures 6A and B are generated from the same original images. (TIF) [file pone.0022703.s003.tif]
